# Multi-level factors influencing the implementation of Cambodia’s National Policy on School Health: a qualitative case study

**DOI:** 10.1186/s41182-026-01103-5

**Published:** 2026-10-29

**Authors:** Saray Pheng, Vandy Tep, Takashi Asakura, Sokhom Chan, Marie Ueno, Noudéhouénou Credo Adelphe Ahissou, Jun Kobayashi

**Affiliations:** 1 Department of Global Health, Graduate School of Health Sciences, University of the Ryukyus, Ginowan, Okinawa 901-2720 Japan; 2 Department of Science, Faculty of Science Education, Battambang Teacher Education College, Battambang City, Battambang Province 020-30304 Cambodia; 3 Battambang Teacher Education College, Battambang City, Battambang Province 020-30304 Cambodia; 4 The Global Collaboration in Nursing Research Division, Shiga University of Medical Science, Ōtsu, 520-2192 Japan; 5 Curriculum and Instruction Unit, Kampot Provincial Teacher Training Center, Kampot City, Kampot Province 070-80100 Cambodia; 6 The United Graduate School of Education, Tokyo Gakugei University, Koganei, Tokyo 184-8501 Japan; 7 Japanese Consortium for Global School Health Research, Ginowan, Okinawa 901-2720 Japan

**Keywords:** Cambodia, Battambang Province, School Health, Implementation factors

## Abstract

**Background:**

Since the 2000 World Education Forum, international organizations have encouraged low- and middle-income countries to strengthen school health systems through national school health policies based on the Health Promoting School (HPS) concept. While these policies offer proven benefits, implementation remains challenging in many low- and middle-income settings. In Cambodia, despite the establishment of the National Policy on School Health (NPSH), implementation remains uneven and insufficient across administrative levels. While implementation factors have been identified at the national, provincial, and elementary school levels, evidence remains limited at the secondary school level. Therefore, the current study investigated the factors influencing NPSH implementation at the public secondary school level in Battambang province.

**Methods:**

This qualitative case study assessed complementary information from 27 key informant interviews (KIIs) with stakeholders involved in policy implementation and 13 documents related to previous implementation efforts. Participants represented national policy, provincial, district, commune, and school perspectives. Content analysis combined deductive coding guided by Vince Whitman’s wheel of factors influencing the implementation of policy and practice, with inductive coding of context-specific themes.

**Results:**

Nine thematic domains were identified to influence NPSH implementation. Policy reform, policy implementation-adapted strategy, and champions and leaders at all levels were described primarily as enabling implementation. Dissemination gaps and limited understanding within the international and national guidelines domain, attention to external forces, and gender gap and mandated roles were described primarily as inhibitors. Dedicated time and resources, administrative and management support, and stakeholder ownership and participation contained both enabling and constraining processes. These categories describe reported implementation experience rather than measured effect or the relative importance of factors.

**Conclusions:**

Participants described an interaction between a binding legal mandate and perceived local autonomy in the implementation of NPSH. Their accounts suggest that a clearer legal framework, support for local adaptation, leadership, and cross-sector participation may help implementation in this setting. This study did not establish causal effects or examine students’ health and learning outcomes. Research across additional provinces, incorporating frontline staff, parents, students, and independent implementation data, is needed to assess the interpretations and their transferability.

## Background

The World Health Organization (WHO) has described schools as major channels for information and an important setting for interventions addressing health-risk behavior among school-age children [1, 2]. The WHO therefore introduced the Health Promoting School (HPS) concept to improve the health and well-being of students, school staff, families, communities, and other stakeholders through coordinated school health activities [3]. The HPS, initiated by the WHO, UNESCO, and UNICEF in 1995, has been influential in the success of primary school health-promoting programs worldwide [4, 5]. In Taiwan, the HPS was initiated in 2002 to improve student and school health, establish a positive school environment, and serve as role models [6]. Also, in Hong Kong, the Healthy School Award is a prime example of how this framework is implemented to enhance students’ health and quality of life [7, 8]. Evidence from many developed countries suggests that the HPS framework can improve educational outcomes, health, quality of life, and economic prosperity, while reducing the burden of disease [9, 10].

To date, Asian and African countries have adopted National School Health Policies based on the HPS concept, including Lao PDR, Nepal, Nigeria, and Kenya [11, 12]. Previous studies have reported that the successful implementation of the National School Health Policy (NSHP) required several factors [13, 14]. Lao PDR showed that comprehensive national planning, well-structured human resource training, and continuous monitoring affected the implementation of the NSHP [11]. Thailand identified seven positive factors influencing NSHP implementation, including strategic alignment, stakeholder involvement, award systems, and sustainable capacity building, as well as negative factors such as unclear administrative roles, instructional limitations, and widespread health challenges among Thai children [15]. Although the HPS framework has been widely adopted worldwide, implementation success varies considerably across countries because implementation is shaped by political, administrative, and socio-cultural contexts [16]. Therefore, understanding context-specific implementation factors is essential for translating national school health policies into effective practice [17, 18].

### Health education in Cambodia

Through hygiene education and the clean-living movement, school hygiene in Cambodia began to shift the primary education curriculum in the pre-1970s, and by then, Science and Hygiene were among the subjects integrated into the curriculum to address poor hygiene and water supply [19]. Influenced by the war between 1970 and 1975, schools were damaged, teachers were killed, formal education ceased, and most of the health service infrastructure was destroyed [20]. To restore educational development, Cambodia worked with development agencies and many non-governmental organizations (NGOs) to build capacity [21, 22]. Students’ health is a crucial factor widely reported to improve the quality of education and increase the graduation rate, as it enhances children’s intelligence and ability to actively engage in educational activities and reduces the dropout rate [23, 24]. These findings provide background but do not establish the effects of school health policy on Cambodian secondary school outcomes. Health issues and malnutrition have been documented as major obstacles to children receiving a better education [25]. Heesch et al. [20] found that children's health status in rural areas of Cambodia is worse than in urban areas [20]. In response to these issues, many nations around the globe, including Cambodia, have focused on enhancing school health by developing National Policies and strategies to increase cooperation among all stakeholders [26]. Also, Cambodia’s Ministry of Education, Youth and Sport (MoEYS) started to formulate health education in the core curriculum in both the primary and secondary school levels, and all components of the health education subject focused on primary health care, mental health, sexual and reproductive health, health and environment, health beliefs, and health economics [27].

### Establishment of National Policy on School Health

Like other Asian countries, Cambodia’s MoEYS established a school health policy in 2006 in response to rapid global change, including industrial advancement, population growth, environmental changes, economic globalization, and changing lifestyles that affect public health [28]. Then, it was implemented in accordance with the proposed MoEYS internal guideline, without a national mandate and cooperation from other ministries. However, the transition from school health policy to the NPSH marked a significant shift in Cambodia's understanding of the global link between education and public health [26]. The Royal Government of Cambodia prioritized school health as an essential foundation for developing human resources, aiming to transform the nation into an upper-middle-income country by 2030 and a high-income country by 2050 [29, 30]. Cambodia’s MoEYS has developed a national action plan to support integrated, multidisciplinary practice in response to the NPSH, serving as a roadmap for implementing school health activities [31]. The collective commitment of various ministries, institutions, development partners, donor agencies, the private sector, and all stakeholders is necessary to develop human resources aligned with global citizenship in the Cambodian context [31].

Following decades of rebuilding the education system after the Khmer Rouge regime, Cambodia reformed its government structure through Decentralization and Deconcentration (D&D) reform policy [32, 33]. Under this framework, educational authority and funding were formally transferred to subnational administration, enabling School-Based Management (SBM) to empower school-level decision-makers to enhance school effectiveness and learning outcomes [32, 34]. Within this evolving context, Cambodia introduced the NPSH to advance educational quality across nine strategic areas, including health services, health education, a safe school environment, nutrition, inclusive learning, and stakeholder collaboration [28, 31]. The NPSH aims to ensure educators are equipped with the skills to teach the prevention of non-communicable diseases (NCDs), deliver essential health services, and cultivate lifelong healthy habits [26]. However, translating the policy from design into widespread practice has faced many challenges. To date, school health programs in Cambodia have focused primarily on primary education, particularly the water, sanitation, and hygiene (WASH) program [35], rather than on the multi-level, multi-sectoral implementation of national policy. This limited execution stems largely from a shortage of dedicated funding [36], passive inter-institutional partnership, and weak inter-ministerial coordination [26, 31].

Currently, the implementation of school health programs in low- and middle-income countries is increasingly important in middle and high schools [37, 38]. Reproductive and mental health issues are global challenges and are considered key areas of health education in middle and high schools [39, 40]. While much research has mostly focused on the implementation of school health programs and the HPS framework globally at the national, provincial, and elementary school levels [35, 41], empirical evidence on the comprehensive implementation of national school health policies remains limited in Southeast Asia, with a gap in evidence on Cambodia’s secondary school implementation [36]. Consequently, it is crucial to identify the factors that facilitate or hinder NPSH implementation across administrative levels, especially at the secondary school level, where adolescent reproductive and mental health needs warrant attention [42]. Therefore, this research aims to investigate the multi-level factors affecting the NPSH implementation in secondary schools in Battambang province, Cambodia, exploring how implementation factors in an understudied setting interact with Cambodia’s distinct legal, administrative, and socio-cultural context, focusing on policy reform, localized implementation strategies, and gender-differentiated roles, which remain limited in Southeast Asia.

## Methods

### Research design

This qualitative case study [43], conducted from August 2025 to January 2026, collected a variety of data to explore a complex phenomenon and key factors across various aspects of NPSH implementation at the provincial, district, and secondary school levels in Battambang Province, using key informant interviews (KIIs) and document analysis. Baxter and Jack (2008) argued that a qualitative case study uses multiple data sources to investigate a phenomenon, revealing multiple aspects from diverse viewpoints and increasing understanding and comprehension [44].

### Study areas

**Fig. 1:**
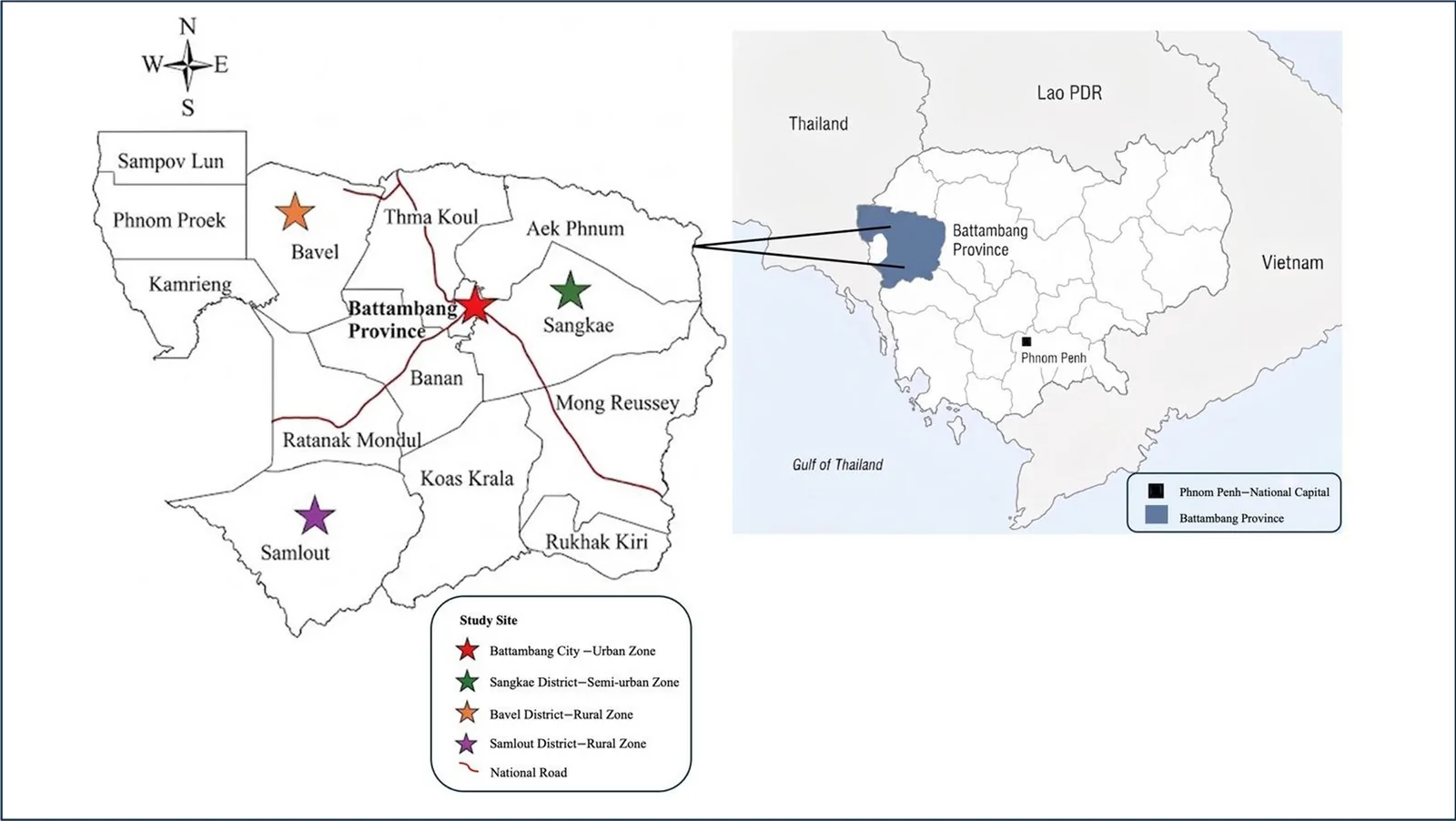
Study sites in Battambang Province, Cambodia. Adapted from the National Institute of Statistics [49]

Battambang Province has been identified as an exemplar and a pioneer in national reform and policy innovation experiments. For instance, recent reforms include the establishment of city councils and one-window services, as well as the testing of policies to address informal settlements [45]. This province implements the School Feeding Program with the support of MoEYS and the World Food Program [46, 47]. It hosts a Teacher Education College, which trains teachers at the primary to lower-secondary levels in accordance with the Ministry’s Teacher Policy Action Plan (2024–2030) and aims to upgrade teacher qualifications and address national teacher shortages [48]. Battambang province was selected as an information-rich case because of its history of educational innovation and collaboration with development partners. This setting allowed exploration of implementation in an information-rich context with a history of policy innovation; its administrative condition may differ from those in other provinces. Four study areas were purposively selected based on the history of NPSH implementation and accessibility to researchers (Fig. 1). The Bavel district (Population: 92,306) is in the northwest, and the Sangkae district (Population: 111,118) is in the northeast. Battambang City (Population: 119,251) is in the province's central part, and the Samlout District (Population: 43,715) is in the southwest [49]. In 2022, the total number of public schools in Battambang province was 1,170, comprising 389 preschools, 654 primary schools, and 127 secondary schools. The number of students enrolled in primary schools aged 6–11 years was 164,335 (78,804 girls), while students enrolled in secondary schools numbered 75,004 (40,550 girls) aged 12–17 years [50]. The data were collected at the provincial, district, and secondary school levels in Bavel and Samlout districts, representing rural zones, and in Battambang City and Sangkae, representing urban and semi-urban zones, respectively.

### Key informant interviews (KIIs)

We conducted KIIs with 27 NPSH implementers (female, *n* = *12*; male, *n* = *15*) involved in the implementation task force. The policy implementers’ task forces comprise policymakers, government administrators responsible for school health activities at school health departments, officers from provincial and district departments, the school management team, and related stakeholders involved in the implementation of school health promotion activities, including NGOs and members of the Commune Committees for Women and Children (CCWC), forming the implementer committees. The key informants (KIs) were selected purposively at each administrative level, and one-on-one interviews were conducted at their offices. We selected two KIs who work as policymakers. Two government administrators from the Department of Education, Youth and Sport, and three participants from three different District Education, Youth and Sport Offices were recruited. Next, one KI from the Department of Health and three KIs from three different Operational Districts (OD) were recruited. Also, we selected two program health officers from two different NGOs whose work focuses on school health promotion at the district level. In addition, five CCWC members were selected from various outstanding communes across the four research sites. At the school level, four model secondary schools (schools that met the five standards criteria of the model school standard assessment) and four non-model secondary schools (schools that have not yet met the five standards criteria of the model school standard assessment) were included. In standards 1 and 4 of the model school standard assessment tools, there are 13 indicators related to school health assessment, and with a passing score across the 5 standards, schools are considered model schools [51]. Finally, nine school principals and deputy school principals from the eight schools were selected for interviews; two participants represented School E (Table 1).

**Table 1:** Characteristics of the 27 key informants

Interviewee code	Gender	Position category	Sector	Qualification	Years in current position
K1	Male	Policymaker	Education	Master’s degree	27
K2	Female	Policymaker	Education	Master’s degree	30
K3	Male	Provincial Officer	Education	Master’s degree	21
K4	Male	Provincial Officer	Education	Master’s degree	18
K5	Female	Provincial Health Officer	Health	Bachelor’s degree	6
K6	Male	District Officer	Education	Master’s degree	34
K7	Female	District Officer	Education	Bachelor’s degree	3
K8	Male	District Officer	Education	Master’s degree	4
K9	Female	OD Officer	Health	Associate degree	35
K10	Male	OD Officer	Health	Bachelor’s degree	30
K11	Male	OD Officer	Health	Master’s degree	6
K12	Female	NGO Program Officer	NGO	Bachelor’s degree	3
K13	Male	NGO Program Officer	NGO	Master’s degree	2
K14	Female	CCWC Member	Community	Secondary School	10
K15	Female	CCWC Member	Community	Secondary School	7
K16	Female	CCWC Member	Community	Secondary School	10
K17	Female	CCWC Member	Community	Secondary School	15
K18	Female	CCWC Member	Community	Primary School	23
K19	Male	Deputy School Principal (School A)	Education	Master’s degree	16
K20	Male	School Principal (School B)	Education	Master’s degree	19
K21	Male	School Principal (School C)	Education	Master’s degree	5
K22	Male	School Principal (School D)	Education	Master’s degree	15
K23	Male	Deputy School Principal (School E)	Education	Master’s degree	9
K24	Female	Deputy School Principal (School E)	Education	Master’s degree	1
K25	Female	Deputy School Principal (School F)	Education	Bachelor’s degree	15
K26	Male	School Principal (School G)	Education	Master’s degree	25
K27	Male	School Principal (School H)	Education	Master’s degree	18

*CCWC* Commune Committee for Women and Children, *NGO* non-governmental organization, *OD* operational district

The interview guideline for the Policy Implementation Assessment Tool for implementers and stakeholders, developed under the United States Agency for International Development (USAID) Health Policy Initiative [52], was adapted and translated from English into Khmer. The original version of this interview guideline comprised seven dimensions of the overall implementation process of health policy, which have been used in health policy analysis in many low- and middle-income countries worldwide [52]. This tool is designed to analyze the operational, political, and cross-sectoral dynamics of policy implementation in low- and middle-income countries (LMICs). This fits Cambodia’s NPSH, which is highly complex and depends on multi-stakeholder involvement in its implementation from the national to the local school level. We adapted this USAID interview guideline as semi-structured open-ended prompts that enable the inductive emergence of context-specific issues. To assess the relevance and clarity of this semi-structured interview guide, a comprehensive discussion and feedback session on the open-ended questions was held with experts from the health and education sectors. Before conducting the main interview, a pilot interview was conducted with implementers of school health promotion programs. Based on their comments and feedback, the semi-structured interview questionnaire was finalized for the main interview. For data collection, the researcher interviewed participants in Khmer for 45–60 min and recorded the interviews using an electronic audio recorder. All 27 participants’ interview responses were then transcribed in Khmer.

### Document review

The document review was conducted to complement information gathered through KIIs. The included documents related to the implementation of this policy in Cambodia include the 2006 version of the school health policy, Annual Progress District Reports, Education Congress Reports, Government Strategy Plans, National Action Plans on School Health, and government remarks at meetings related to school health (Table 2). The information in those documents focuses on the implementation process and strategies, the policy guidelines, and the implementation progress report. The required documents were provided by the implementer at all levels and made available to the investigator via the *Telegram* platform and through searches on government and ministry websites.

**Table 2:** Documents reviewed for analysis of the national policy on school health implementation

No	Name of the documents	Institution	Year of publication
1	Pentagonal Strategy-Phase I	Royal Government of Cambodia	2023
2	Remarks by Prime Minister: Introducing the “Pentagonal Strategy–Phase I”	Royal Government of Cambodia	2023
3	School Health Policy	Ministry of Education, Youth and Sport	2006
4	National Action Plan on School Health 2021–2030	Ministry of Education, Youth and Sport	2022
5	Education Strategic Plan 2019–2023	Ministry of Education, Youth and Sport	2019
6	Education Strategic Plan 2024–2028	Ministry of Education, Youth and Sport	2024
7	Report on Education Congress Battambang 2021–2022	Provincial Department of Education, Youth and Sport	2023
8	Report on Education Congress Battambang 2022–2023	Provincial Department of Education, Youth and Sport	2024
9	Report on Education Congress Battambang 2023–2024	Provincial Department of Education, Youth and Sport	2025
10	Report on Education Congress Battambang 2024–2025	Provincial Department of Education, Youth and Sport	2026
11	Policy and Action Plan on School Health 2023–2028	Provincial Department of Education, Youth and Sport	2022
12	Achievements in the Implementation of the National Action Plan on School Health 2021–2025	Provincial Department of Education, Youth and Sport	2026
13	Summary Report on the Evaluation of Education, Youth, and Sport Work for the Bavel District Administration for the 2024–2025 School Year and Directions for the 2025–2026 School Year	District Education, Youth and Sport Office	2026

### Data analysis

This study employed content analysis using the wheel of factors influencing implementation of policy and practice (adapted from Vince Whitman) [53]. The 12 factors are (1) vision and concept/international and national guidelines; (2) dedicated time and resources; (3) stakeholder ownership and participation; (4) team training and ongoing coaching/learning community; (5) cross-sector collaboration; (6) champions and leadership at all levels; (7) data-driven planning and decision-making; (8) administrative and management support; (9) adapting to local concerns; (10) attention to external forces; (11) critical mass and supportive norms; (12) stage of readiness [53]. The USAID assessment tool informed the interview prompts used for data collection, whereas the twelve factors recommended by Vince Whitman provided the theoretical lens for deductive analysis. Open-ended prompts and inductive coding allowed issues outside the predefined categories. All 12 factors of Vince Whitman were initially considered for deductive coding. First, the KIs’ responses and the document review were coded in Khmer, and the Khmer text was then translated into English using an AI-based translation tool (Gemini, Google). Before translation, all transcripts and documents were de-identified, and only the de-identified text was entered into the AI tool. All translations were verified against the original Khmer by two researchers. Second, interview and document data were categorized using a hybrid deductive-inductive coding scheme to identify reported supports and barriers. To ensure accuracy and quality, four researchers conducted the data analysis and consulted regularly with other members of the research team.

### Trustworthiness of the study

To ensure the trustworthiness of this study, several procedures were used to support credibility and consistency. At the end of the interviews, the researcher shared a summary with the participants and invited corrections and clarification. Related implementation documents were obtained to complement interview accounts and reviewed by the research team. During data analysis, two researchers independently coded a subset of the Khmer transcripts and developed a shared codebook and initial categories. Two additional researchers then reviewed the codes and category structure. To maintain consistency, the four researchers discussed differences in interpretation and refined the coding through consensus meetings as analysis continued. Finally, to support transferability, we shared the study context, including the research site, information profiles, analysis methods, and findings, which were evaluated collaboratively by the research team to ensure relevance to the Cambodian context.

## Results

Nine thematic domains were identified in accounts of NPSH implementation across provincial, district, and school levels. Three inductively developed categories were identified through inductive analysis, which reflect the context-specific findings in this study: policy reform, policy implementation-adapted strategy, and gender gap and mandated roles. Six categories were identified through deductive analysis using the wheel of factors influencing implementation of policy and practice (adapted from Vince Whitman), such as dedicated time and resources, administrative and management support, stakeholder ownership and participation, champions and leaders at all levels, attention to external forces, and international and national guidelines. The domains describe implementation issues; processes within each domain were interpreted as enablers, inhibitors, or both, depending on context.

### Enablers

#### Policy reform: perceived implications of the transition from guidance to mandate

Participants perceived the transition from the 2006 ministerial-level guidance to the 2019 national policy as facilitating implementation. One policymaker described spending time and effort persuading other ministries to participate before the reform, and reported that the national mandate subsequently made coordination and mobilization easier. These accounts reflect perceived changes in implementation processes rather than an independent assessment of the reform’s effects.

“*Now that this policy reform is in place, implementation has become much smoother. By simply referencing the government's official directives, we can quickly mobilize other ministries and stakeholders. We’ve observed a high level of flexibility and rapid action because everyone is already in agreement and working toward the same goal. In the past, there was some friction; just wanting to do one thing after another required walking around lobbying, walking around telling people why we have to do that thing, whether they would agree with us or not...”*

*(K1, Policymaker)*

The reform of this policy aligned closely with the Royal Government’s Rectangular Strategy Phase IV, which aims to develop high-quality, productive, and healthy human resources to drive Cambodia toward its vision of becoming an upper-middle-income country by 2030 and a high-income country by 2050. With this clear vision, the national Action Plan on School Health 2021–2030 was established in accordance with Cambodia’s Education 2030 Roadmap and the 2030 Sustainable Development Goals.

*“…acknowledging that Cambodia is committed to becoming an upper-middle-income country by 2030, we elevated the ministry-level policy to a national policy in 2019 to encourage all learners to achieve better health and learning outcomes and to promote better human resource development by 2030.”*

*(K2, Policymaker)*

#### Policy implementation-adapted strategy

Policy implementation-adapted strategy is an inductive category identified in this study. The two subcategories, for example, (i) provide a legal framework mechanism, and (ii) autonomy in the implementation process, were described to support the implementation of this policy. At the provincial and school levels, the legal framework mechanism effectively supports the implementation of national policy by defining cooperation and tasks of relevant stakeholders. At the grassroots level, such as in schools, they cannot arbitrarily enforce health rules, such as banning unhealthy food vendors or initiating health curricula, without an official legal mandate. The legal framework protects school principals and local implementers by giving their actions legal weight and public recognition. Two key informants stated that a strong legal framework supports the development of a culture of work, and that this strategy is part of the approach to motivating people to get involved. The following example of quotes illustrates their experience of legal framework support for the policy implementation:

*“…to ensure that health initiatives operate smoothly, we established a clear strategy for the path forward and supported people, particularly legal support (meaning the regulatory framework). This involves preparing and disseminating various legal instruments and standards, managing them effectively, and implementing national policy efficiently. Without these foundations, it would be extremely difficult for us to achieve our goals.”*

*(K4, Provincial Officer)*

*“Public school activities required acknowledgment from authorities, but when official circulars are issued from the top, they possess a formal quality. For public schools in local areas, it makes our actions legal and publicly recognized, which gives our school the capacity to implement them well…, schools cannot do anything without legal norms or circular guidelines.”*

*(K22, School Principal)*

Participants described autonomy as giving local leaders greater discretion to innovate and manage available resources. The two participants mentioned that the MoEYS has adopted an open-minded approach, allowing subnational governments to innovate in school development and adapt financial management to local circumstances.

*“The Minister has adopted an open-minded approach, granting me the autonomy to innovate and implement new initiatives. A prime example of this is the 'School Zone' project, which was developed under my own personal initiative. We have granted educational institutions autonomy regarding financial management and resource mobilization through School-Based Management (SBM).”*

*(K3, Provincial Officer)*

*“…by developing our own strategic and action plans—while remaining aligned with national policy—we gain the flexibility to execute tasks according to local needs and demonstrate concrete results to the national level. This autonomy is far more effective than a rigid, top-down mandate, as it fosters greater ease and ownership in project execution.”*

*(K4, Provincial Officer)*

#### Champions and leaders at all levels

Two main subcategories were identified: strong leadership and the establishment of the committee at all levels. KIs argued that effective, strong leadership is paramount in policy implementation, a prerequisite for organizational success, stability, and long-term sustainability of implementation, involves active monitoring, and identifying gaps. The stability and long-term sustainability of policy implementation are fundamentally rooted in leaders' professional ethics and transparency. Following is an example quote:

*“Generally, we operate within the Ministry’s national structure; however, at the provincial level, we have the Governor and the Provincial Department of Education. Therefore, each relevant institution must exercise strong leadership to ensure the policy's successful implementation.”*

*(K3, Provincial Officer)*

*“Participation and trust in the leadership of the principal is first, second is the transparency of the principal, and third is stakeholder involvement... Transparency includes the budget and everything in the school… if leaders dare to be dishonest with the teachers in the school, it will not work.”*

*(K6, District Officer)*

Implementing the policy requires involvement from all relevant stakeholders at all levels. In the Cambodian context, establishing the school health committee as a structured hierarchy ensures that the policy does not remain isolated at the national level and defines specific roles and responsibilities to enhance implementation effectiveness. At the provincial and district levels, the school health committee comprises diverse stakeholders, including local authorities, educational institutions, health centers, parent representatives, and community members, and is chaired by the provincial governor. At the grassroots level, School Management Committees and Class Management Committees are the policy enforcers.

*“…, 23 ministries are implementing this policy at the national government level, so we have a National Committee on School Health, an inter-ministerial committee... and at the sub-national level, there are school health committees of the capital/province, city/district/khan, and school health committees of the educational institutions. There is a structure that must be implemented from top to bottom.”*

*(K1, Policymaker)*

*“Currently, an Inter-Ministerial Committee for School Health has been established, involving numerous ministries and public institutions. The composition of the School Health Committees includes representatives from district referral hospitals and local health centers.”*

*(K4, Provincial Officer)*

*“…we have instructed all schools to establish School Management Committees and Class Management Committees in all schools... They have another role: they must print a banner related to controlling what the Ministry of Education allows to be sold in schools, so they must print that banner and place it in front of the vendors.”*

*(K6, District Officer)*

### Inhibitors

#### International and national guidelines

Dissemination gaps and limited understanding were identified as barriers within the international and national guidelines domain. The concern was how guidance was communicated and understood, rather than the guidelines themselves. Due to the limited budget, policy dissemination relies heavily on cascade training or forwarding documents with limited orientation. Participants perceived a risk that the core message could change as information moved through the administrative hierarchy. One policymaker reported that subsequent dissemination could depart from the concepts presented in training.

*“The challenging problem is that when those who joined the orientation and the training of policy implementation and went out to disseminate, the results obtained differ from our expectations… because they didn’t fully understand what they learned, and the dissemination strays far from the original concepts…, so this is the point we worry about the most.”*

*(K1, Policymaker)*

Participants described ambiguity about how much implementation was expected and a limited explanation of school food safety guidance. The gap in dissemination also affects environmental regulation within the school. One district officer reported that some school principals and vendors did not understand or read the guidance. These accounts indicated gaps in dissemination and understanding. They do not establish that the food sold in schools was toxic or that all principals regarded the policy as optional.

*“By ambiguous, I mean that firstly the higher-ups still use the phrase 'implement according to your capacity'..., but to dictate exactly how much to implement, that hasn't been officially released yet…”*

*(K27, School Principal)*

*“When asking the vendors, they say they didn't know. When asking some principals, they say they don't understand yet..., for example, they didn't go to learn; when they drop tasks off with us, we just read them, then we pass them on to the principal. Sometimes, the principals don't even read the guidelines”*

*(K7, District Officer)*

#### Attention to external forces

In this category, “Attention to external forces”, expressed the existence of hardship and challenges in the operational area. Participants described four main external pressures that constrained policy implementation: border conflict between Cambodia and Thailand, transportation and geographic isolation, natural disasters, and household socio-economic pressures. The conflict disrupts policy implementation primarily by creating severe security concerns that restrict the mobility of frontline implementers and competing demands on local resources. Local communes are forced to redirect their limited administrative and development funds to support refugees, leaving local health and capacity-building programs severely underfunded. One CCWC member described residents preparing to evacuate, and her own reluctance to travel far to work.

*“...because our country has a border conflict with Thailand..., our citizens have prepared to evacuate..., as for me, I am also cautious and do not dare to travel far from home for work.”*

*(K18, CCWC Member)*

Participants reported that geographical isolation, poor transportation infrastructure, and limited telecommunications complicated outreach and delayed responses to urgent situations. The frontline worker faces massive challenges navigating mountainous terrain and crossing streams. The vast distance between villages and the lack of telecommunications networks in remote areas mean that commune workers, police, and health officers cannot coordinate effectively to arrive at incident scenes or deliver health services simultaneously. These accounts suggest a barrier to timely joint response and access to services in some locations.

*“Regarding transportation, our citizens often reside in remote areas with long or difficult travel routes. Some must cross rivers, while others are preoccupied with agricultural labor or work as migrant workers deep within the forests.”*

*(K9, OD Officer)*

*“…, another problem is cooperating with local police: because we are far apart (20 to 30 kilometers), communication is difficult, and it's not easy to arrive at the scene together. Because some places have no phone signal service, we cannot intervene in time...”*

*(K18, CCWC Member)*

Participants reported that natural disasters, particularly heavy rain and flash floods, disrupted outreach and intervention activities. Participants described some rural roads as impassable during severe weather. Health officers and commune representatives report being unable to cross flooded areas to deliver health education or rescue vulnerable individuals. These reports indicate disruptions in particular circumstances rather than the complete suspension of all outreaches.

*“Geographical locations, border disputes, and natural disasters such as rain and flooding hinder our progress and prevent us from intervening on time, especially when the roads are difficult to travel.”*

*(K18, CCWC Member)*

*“Going down there, the roads are very difficult, up and down, and suppose when I go, it hits the rainy month, when it rains heavily, we cannot cross”*

*(K5, Provincial Health Officer)*

Household socio-economic pressure heavily influences policy implementation at the commune and school levels. Key informants linked household financial pressures to difficulties supporting children’s schooling and purchasing hygiene supplies. One school principal reported that agricultural market problems, debt, and perceived inability to afford sanitary pads were possible contributors to embarrassment and school dropout among female students. Economic hardship forces parents to migrate across borders to other provinces for work, taking their children with them and stopping their education entirely. This study did not assess absenteeism, school dropout, or students’ learning outcomes, nor did it establish a causal relationship between these pressures and those outcomes.

*“Regarding debt, it also has some connections. For example, a student's parents grow cassava, but when they cannot find a market for it, it undermines their ability to support their children's schooling. When the children need to buy hygiene products, they may not be able to afford them. Sometimes, female students feel ashamed when they don't have money to buy sanitary pads..., this can be a minor factor that causes students to drop out of school.”*

*(K26, School Principal)*

Participants described competing livelihood demands that could limit participation in preventive health and health education. A health worker reports that livelihood demands are limiting some parents’ participation in vaccination and health education activities. This report reflects a perceived competing demand on household time rather than evidence that parents generally disregard their children’s health.

*“Another thing is they think about their livelihoods, thinking about earning a daily living, forgetting about their children. Whether the child is sick or sometimes forgets the day and month for vaccinations…, they are so busy with their occupations that they forget to think about health…”*

*(K9, OD Officer)*

“*Another thing is the economy of our district; people do not have much work. When citizens do not have jobs, it can be difficult for them, leading them to migrate. When they migrate for work, it weakens the data of participants joining us (health outreach), so we do not get much positive impact...”*

*(K12, NGO Program Officer)*

#### Gender gap and mandated roles

All CCWC members interviewed in the present study reported the same issue regarding the gender-sensitive tasks involving women’s and children’s health and safety. Members of the Commune Committee for Women and Children (CCWC) are elected to their positions every 5 years. Participants reported that women experiencing domestic violence or sexual abuse preferred to discuss sensitive experiences with female staff and that male colleagues were less comfortable asking detailed questions. One participant explained this by the fact that most of the cases she handled involved girls and women. These accounts suggest that gender and trust shape access to support in this setting.

*“Men cannot resolve conflict issues; only we women can help because men do not dare to ask female or child victims in-depth questions as women do. When a woman suffers from sexual violence, men do not dare to ask...”*

*(K17, CCWC Member)*

*“When women face problems, they do not dare to go and discuss them with the male commune council members. So, it is only us women; they come to us and can talk about their internal problems, and they dare to tell us their hardships”*

*(K14, CCWC Member)*

CCWC members faced the challenge of the devaluation of their roles while working in the community. Following is the quotation of one key informant, who reported the gender gap and the undervaluation impact on women’s working experience:

*“Another challenge in my role is that they do not give much value or 100% rights to women because some people still hold the old mindset that women do not need to be highly educated and cannot do big tasks. There is still a gender gap in fulfilling tasks. However, even if they do not value me, I continue my work without giving up, even though I sometimes feel resentful”*

*(K18, CCWC Member)*

### Factors with dual effect (enabler and inhibitor)

#### Dedicated time and resources

Human resources and three additional inductively developed subcategories observed within the domain, such as persistence and self-resilience, flexibility and adaptiveness, and resource mobilization, were identified under the dedicated time and resources domain. Human resource constraints critically hinder policy implementation. Participants report that the delays in staff recruitment and a lack of specialized health teachers have created a chronic staffing shortage. This shortage of human resources means that health education classes are either skipped, poorly integrated, or managed by exhausted staff who lack the technical expertise to deliver the curriculum effectively. Participants described shortages of personnel responsible for health rooms and health education.

*“We cannot implement it successfully because we do not have a responsible person stationed at the health room all the time, and the lack of teachers heavily impacts it. Without teachers, nothing can be done; we need core teachers who can teach health education…”*

*(K23, Deputy School Principal)*

Persistence and self-resilience influence implementation by ensuring that implementers effectively maintain their roles throughout grassroots implementation and in situational adaptive strategies. This concept describes the behavioral adaptation observed among frontline implementers as they negotiate daily challenges in their fieldwork. It shows the motivation of local actors who maintained their efforts despite insufficient support and resources. The following quotations illustrate participants’ accounts of persistence and self-resilience:

*“When we negotiate the budget with the Ministry of Finance, we put in a lot of effort. We remain resilient and never give up; if a document is missing evidence, we continually seek and add more until it is persuasive.”*

*(K1, Policymaker)*

*“Another challenge in my role is that they do not give much value or 100% rights to women..., however, even if they do not value me, I continue my work without giving up, even though I sometimes feel resentful.”*

*(K18, CCWC Member)*

Participants described flexibility and adaptation as helping local actors fit activities to local circumstances and community members’ availability. Adjusting meeting times and locations was perceived as supporting participation and ensuring that health outreach reaches the target audiences.

*“We maintain a high degree of flexibility and adapt our approach based on the specific needs and evolving contexts of each situation.”*

*(K16, CCWC Member)*

*“Some citizens are market vendors, so even at 12 PM or during their rest hours, I must go meet them, giving them the right to choose the time and place. If we set the time for an 8 AM meeting, absolutely no one will come...”*

*(K17, CCWC Member)*

To address the budget shortage from the central state, stakeholders at the community and school levels deploy highly innovative resource mobilization strategies. Participants described mobilizing contributions from parents, communities, NGOs, and private donors to supplement limited public resources. When teachers, principals, and frontline health workers exhibit strong professional dedication, passion, and commitment, they can single-handedly drive policy forward and overcome material and financial constraints. These accounts suggest that commitment can support resource mobilization, but do not establish that individual effort can overcome structural resource constraints.

*“Upon receiving reports from the school principal, the CCWC mobilizes resources from partner NGOs… I initiated a project to construct a local school. I coordinated with partner NGOs, private donors, and community members, mobilizing their support to make the construction of the school a reality.”*

*(K18, CCWC Member)*

*“The schools that we see can develop, have beautiful buildings, a clean environment, and clean toilets...we do not use this Program Budgeting fund; we use funds mobilized from parents through the establishment of School Management Committees.”*

*(K3, Provincial Officer)*

#### Administrative and management support

Within administrative and management support, key informants reported that weaknesses in monitoring and evaluation were a barrier, and that routine progress reports supported communication with higher administrative levels. Participants reported a lack of formal monitoring and evaluation mechanisms, as well as oversight of the policy’s implementation, at the provincial and local levels. Participants perceived the absence of oversight and supervision from higher levels as barriers to translating policy into practice.

*“To date, I have observed that there has been an absence of high-level ministerial oversight or formal evaluation regarding the implementation of this policy at the provincial and local levels.”*

*(K4, Provincial Officer)*

*“Health education teaching seems not to have been monitored yet...I noticed that the office does not yet have specialized officers in health education either, so if they come down to observe the teaching, they don't know how to observe or what feedback to provide.”*

*(K26, School Principal)*

Although oversight, monitoring, and evaluation are lacking, implementation needs to continue. Regarding information sharing with higher administrative levels, the grassroots implementer regularly updates their progress through reports.

*“The physical monitoring doesn't exist. We only report to higher levels, including the district CCWC and provincial CCWC, because our chain of command goes there... Yes, we send reports every month.”*

*(K15, CCWC Member)*

*“Upper-level takes reports every month. Those who implement the policy send reports monthly, quarterly, 6 months..., we collect reports from relevant institutions...we summarize them and send them to the upper level.”*

*(K4, Provincial Officer)*

*“..., we summarize all of the progress reports for the top leaders so they can see the big picture. When they see the facts, they can decide how much more money and support they need to invest in schools to make the national plan a success.”*

*(K2, Policymaker)*

#### Stakeholder ownership and participation

Three subcategories, including limited stakeholder engagement, multi-stakeholder participation, and synergistic partnership, were identified within this domain. A community member attributed limited engagement among some residents to low health literacy and awareness. This phenomenon is described below:

*“The problems we face when we go down conducting health outreach are: firstly, a small number of citizens do not understand health; secondly, they don't participate much with us; thirdly, as they say, they have little knowledge, they don't understand much about the dissemination provided to them, they think it has no benefit.”*

*(K14, CCWC Member)*

Among the four areas, one district found that implementation success was due to a strong, synergistic partnership and multi-stakeholder participation that led to a significant transformational outcome. One district education officer attributed perceived implementation achievement as follows:

*“Our district has achieved a lot of success… because of the high level of professional dedication from our teachers, we maintain consistent communication with the Class Management Committees, and strong parental involvement. The successful implementation in our district is attributed to our high-quality teaching staff, excellent school facilities, and a significant number of dedicated principals. We have a public service champion school ranked 7th nationwide”*

*(K6, District Officer)*

## Discussion

**Fig. 2:**
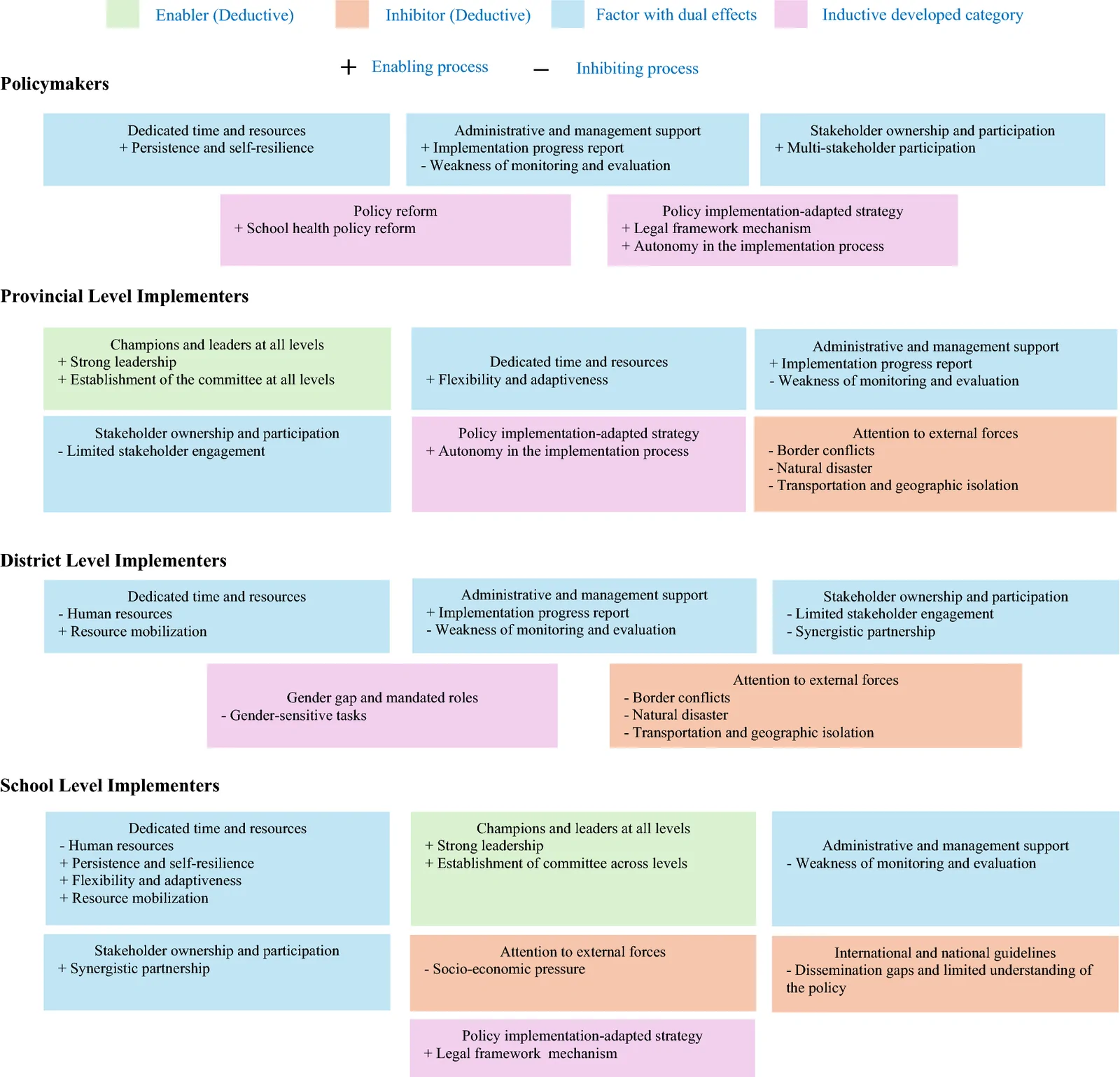
Reported factors in national policy on school health implementation across administrative levels

This study identified nine thematic domains shaping NPSH implementation. Policy reform, policy implementation-adapted strategy, and gender gap and mandated roles were developed inductively. These categories were not explicitly reported as separate categories in the selected comparator studies previously conducted in Thailand, Lao PDR, and Indonesia [11, 15, 35]. This comparison does not establish that the underlying concepts are new to implementation research or absent from all Southeast Asia’s previous studies. The contribution is a context-specific account of the formal transition to national policy, the use of regulatory support alongside local autonomy, and the gendered duties among community members. These categories overlap conceptually with some factors recommended by Vince Whitman, including adapting to local concern, cross-sector collaboration, and leadership. These categories should be interpreted as analytical elaboration rather than proof of deficiencies in the framework. Six other domains were mapped onto Vince Whitman’s original deductive analysis, including champions and leaders at all levels, international and national guidelines, attention to external forces, dedicated time and resources, administrative and management support, and stakeholder ownership and participation. Among these nine thematic domains, three function primarily as enablers, three as inhibitors, and three have dual effects, acting as enablers and inhibitors depending on the administrative level and local context in the area of operation (Fig. 2).

### Key enablers in policy implementation

Participants described policy reform as an important enabling condition for implementing NPSH. Participants perceived the national policy as providing greater authority for inter-ministerial coordination than the earlier ministerial guidance. Interministerial participation is essential to a mandatory obligation that effectively empowers sub-national implementers in the Cambodian context. In the present study, the perceived authority of national policy appeared to be relevant, along with its technical content. This finding is consistent with Walt and Gilson's observations, which reported that changes in policy content fundamentally reconfigure power dynamics, official duties, and the commitment of actors responsible for its implementation [54]. It also reflects guidance calling for joint activities across multiple sectors at all levels of government toward the same goal, and commitment to making every school a health-promoting school [5]. Reforming the NPSH aligns with the Royal Government’s Rectangular Strategy Phase IV, which aims to develop human resources to achieve upper-middle-income status by 2030 and high-income, developed-nation status by 2050. Aligning school health policy with ongoing education strategy and standards yields the greatest success; for instance, integrating the NSHP into the school management action plan and the core curriculum enables instructors to adopt health-promoting practices as part of their daily routines [15]. While previous studies emphasized leadership, resources, stakeholder participation, and administrative support, our findings suggest that transforming a ministerial guideline into a legally mandated national policy fundamentally reshaped inter-ministerial accountability and implementation processes. Nevertheless, retrospective accounts do not isolate the effect of the policy reform from changes in leadership, resources, or administrative arrangement. Policy reform is therefore a plausible implementation consideration in this setting rather than a demonstrable cause of improved performance.

Key informants describe regulatory support and local autonomy as complementary aspects of implementation. While the national level determines the regulations and policies, the subnational level must be flexible in implementing the legal framework and in supporting local schools [55]. Policy implementation demands multi-sectoral involvement; thus, legislative measures can provide a strong, binding framework to support the enforcement of mandates and accountability [56]. Ogasawara et al. [16] reported that the government of the Lao PDR issued a decentralization decree that shifted decision-making and financial management to subnational administrative officers, who should be able to follow the guidelines independently [16]. Our findings illustrate a critical balance between centralized policy mandates and subnational operational autonomy. While top-down national reform provides the legal framework and inter-ministerial accountability across Cambodian ministries, local autonomy and SBM allow educators to adapt policies to local constraints. However, this study did not confirm that autonomy resolves resource shortages. Clear responsibilities, adequate resources, and accountability remain critical for effective adoption.

Participants emphasized strong leadership commitment, transparency, and the establishment of multi-level committees to support school health activities. A key factor in the successful implementation of school health policies is leadership commitment, and the proactive skills of the school principal are critical for resource mobilization and prioritizing support for school health activities [16, 57]. In this study, leadership was described across several administrative levels, suggesting that implementation depends on sustained alignment among national, provincial, district, and school actors. To support these leaders, operational committees anchor governance and accountability. An operational framework updated by WHO reported that the standard educational environment in an authentic health-promoting school demands explicit administrative leadership support and the establishment of multi-sectoral committees to anchor accountability [58]. At the provincial level, school health committees comprised government officials, representatives from the health and private sectors, community members, authorities, and parents to participate in implementation. Previous research has shown that the school’s multidisciplinary wellness team and local school health committees, which consist of social workers, counselors, nurses, doctors, teachers, parents, students, and community members, are described as supporting implementation [16, 59]. However, while supporting these structures facilitates coordination, their presence alone does not ensure effectiveness or sustainability.

### Key inhibitors in policy implementation

NPSH implementation is highly vulnerable to macro-level external factors, including border conflicts, natural disasters, isolated geography with poor transportation and infrastructure, socio-economic pressures, the gender gap, and mandated roles. In December 2025, the WHO situation analysis reported extensive school closures and disruptions to health services during the Cambodia–Thailand border conflict [60]. Schools in remote or conflict-affected areas suffer from stark infrastructure disparities, and crises often strip health programs of their priority status, disrupting the delivery of essential health services [4, 61]. Beyond regional crises, household-level constraints substantially weaken program delivery. Socio-economic pressures within households were a primary obstacle to the reach of school-level programs. The evidence from reviews, surveys, and qualitative studies shows that low socio-economic pressures within households and migration-related livelihood pressures reduce school attendance and educational continuity [62, 63]. Consistent with this evidence, participants linked difficulties in obtaining hygiene supplies, agricultural livelihood problems, and labor migration to barriers to schooling and to participation in outreach. Notably, these accounts reflect perceived barriers to the program’s reach rather than a direct evaluation of its effect on student well-being or learning outcomes.

Accounts from CCWC participants revealed noticeable gender gaps and mandated roles, especially through reports of sensitive casework and the perceived undervaluation of female staff. WHO explicitly emphasized that gender inequality significantly affects both educational and health outcomes [61]. In line with this, our findings suggest that policy implementation is rarely gender-neutral in practice, even when the policy text itself remains silent on gender. Overlooking this dimension risks placing an undue operational burden on a narrow group of female implementers. While these results suggest that implementation duties may be distributed unevenly along the gender lines, they do not indicate any inherent differences in staff capacity.

Participants described gaps in dissemination and understanding as guidance moved through administrative levels from the national ministry to provincial and district levels. When there is a failure to promote awareness and effective translation into practice, school staff and local implementers face role uncertainty and a lack of unified perception, leading to inconsistent application [58].

### Factors with dual effects

Key informants reported staffing and funding constraints alongside efforts to adapt activities and mobilize additional resources, mirroring challenges observed in other school health settings [64, 65]. To address these challenges, the findings emphasized that implementer persistence and self-resilience, adaptive flexibility, and resource mobilization are essential for overcoming systemic barriers and sustaining implementation. Hung et al. [66] emphasized that resilience among school staff facing heavy workloads is crucial, given significant time constraints and competing priorities [66]. Thus, to overcome those trials without losing enthusiasm, implementers need internal motivation and empowerment from school management, and it is necessary to build the head teacher's capacity to ensure teachers’ motivation and self-efficacy [67]. Furthermore, flexibility to meet specific needs and the use of an adaptive strategy to local concerns are also crucial to the effectiveness of school health implementation, especially during pilot programs [11, 57, 66]. When state budgets are inadequate, schools must proactively rely on staff resilience and innovative resource mobilization to sustain their initiatives independently [66]. This study shows that the grassroots implementer mobilized resources from the community, private partners, and donors to sustain policy implementation. One key success factor for implementing NSHP in Thailand is effective fundraising, and the school engages with private companies, temples, the community, and local municipalities to mobilize financial and technical resources [15]. However, reliance on voluntary contributions may vary across schools and communities. Predictable financing and adequate staffing remain a necessary consideration for sustainable implementation.

Administrative and management support, as well as stakeholder ownership and participation, demonstrate dual effects, acting as both enablers and inhibitors. Participants described limited direct monitoring alongside routine reporting to higher administrative levels. Weaknesses in real monitoring and evaluation significantly undermine policy implementation [4]. When administrative roles are vague or lack supervision, this disconnect between policy and practice hinders implementation [15], leading to fragmented implementation and a lack of accountability [64]. However, the regular use of an implementation progress report provided intermediary results and crucial feedback that motivated local implementers [59] and narrowed the perception gap between national authorities and the school level [11]. The findings also showed that limited stakeholder engagement undermines the sustainability of policy implementation. However, fostering synergistic engagement among local communities, parents, NGOs, and businesses can overcome budget constraints and promote a deeply ingrained culture of health [57].

This study suggests that successful policy implementation in Cambodia may benefit from multi-ministerial involvement, institutional accountability, and mandatory accountability. First, because NPSH already exists, the priority is to clarify and operationalize responsibilities under that policy, rather than recommend its reintroduction. Second, local adaptation should be supported within clear accountability arrangements with attention to staffing, financing, geography, and infrastructure [68–70]. Third, establishing school health committees at all administrative levels could be supported by clearly defined responsibilities and coordination across levels [11, 57, 58]. Fourth, operational guidance should ensure the equitable distribution of sensitive casework and provide dedicated support for staff handling gender-responsive tasks. Fifth, policy dissemination should include opportunities to verify understanding and obtain practical feedback at the grassroots levels. Sixth, implementers' involvement in proactive resource mobilization is a crucial component of sustainable school health initiatives. WHO recommends the same strategy to raise funds and mobilize resources from authorities, social organizations, and former partners, including non-governmental actors, to address financial barriers [71]. Finally, the routine implementation reporting should be combined with administrative supportive supervision and ongoing feedback. These recommendations serve as a basis for consideration and evaluation, rather than as interventions demonstrated in this study to improve outcomes.

While this research offers comprehensive insights into the operational realities of policy implementation, it has several limitations. First, this study purposively selected an information-rich case in one province. Site accessibility, the history of implementation and inclusion of model and non-model schools, and selection of communes described as outstanding may favor settings with established activities or strong networks. This case is not representative of the whole nation of Cambodia, and the assumption that Battambang province provides optimal conditions was not tested. Transferability depends on similarities in governance, resources, and exposures to conflicts and disasters. Secondly, this study explored implementation processes through interviews and documents, which did not measure the actual epidemiological, student health, attendance, school dropout, or academic outcomes. This research cannot establish the causal effects of policy reform or implementation strategies. Third, the accounts of policy implementers may reflect recall and social desirability bias, particularly regarding self-reported compliance and achievements. Related documents provide a complementary perspective but may also reflect institutional reporting priorities rather than independent verification. Fourth, the key informants included in this study were policymakers, administrators, school management leaders, NGO officers, and CCWC members. However, it did not capture the perspectives of parents, students, or classroom teachers as distinct participant groups. Future research should consider triangulating implementer accounts with independent monitoring data, extending this multi-level design to additional provinces with different risk-area profiles, and broadening the perspective to multiple implementers who experience NPSH implementation directly. Student health and learning outcomes should be assessed to evaluate the overall effectiveness.

## Conclusions

This study identified nine thematic domains in accounts of the implementation of Cambodia's NPSH in public secondary schools in Battambang province. Six domains corresponded to factors in Vince Whitman’s wheel, and three were developed inductively and reflect the Cambodian context: policy reform, policy implementation-adapted strategy, and gender gap and mandated roles. Participants perceived the transition from ministerial guidance to national policy as facilitating inter-ministerial coordination and accountability, while local autonomy was perceived as allowing provincial and school-level actors to adapt implementation to local conditions. These findings highlight the potential importance of considering the interaction between national legal frameworks and local implementation autonomy when examining school health policy implementation. Future research across different provinces and implementation contexts is needed to determine the transferability of this finding and its relation to student health and academic outcomes.

## Data Availability

The data are not publicly available because they contain potentially identifiable qualitative interview material. Reasonable requests may be considered by the corresponding author, subject to ethical and institutional approval.

## Abbreviations

CCWC: Commune Committee for Women and Children
HPS: Health Promoting School
KIs: Key informants
KIIs: Key informant interviews
MoEYS: Ministry of Education, Youth and Sport
NCDs: Non-communicable diseases
NGOs: Non-governmental organizations
NPSH: National Policy on School Health
NSHP: National School Health Policy
OD: Operational district
SBM: School-based management
UNESCO: United Nations Educational, Scientific and Cultural Organization
UNICEF: United Nations Children's Fund
USAID: The United States Agency for International Development
WHO: World Health Organization
